# High spatial and temporal myocardial CINE T2* mapping at 7.0 T: a feasibility study

**DOI:** 10.1186/1532-429X-15-S1-W29

**Published:** 2013-01-30

**Authors:** F Hezel, C Thalhammer, S Waiczies, J Schulz-Menger, T Niendorf

**Affiliations:** 1 B.U.F.F., MDC Berlin, Berlin, Germany; 2 Experimental and Clinical Research Center, a joint cooperation between the Charité Medical Faculty and the Max Delbrueck Center for Molecular Medicine Campus , Berlin, Germany; 3 Department of Cardiology and Nephrology, HELIOS Klinikum Berlin Buch, Berlin, Germany

## Background

Myocardial tissue characterization using T2* relaxation mapping techniques is an emerging application of clinical cardiovascular magnetic resonance imaging. The increase in microscopic susceptibility at higher magnetic field strengths renders myocardial T2* mapping at ultrahigh magnetic fields conceptually appealing. This work demonstrates the feasibility of myocardial T2* imaging at 7.0 T and examines the applicability of temporally-resolved and high spatial resolution myocardial T2* mapping.

## Methods

Imaging was conducted using a 7.0 T whole body MR scanner (Magnetom, Siemens Healthcare Erlangen) together with a 16 channel TX/RX coil array on 8 healthy subjects without any known history of cardiac disease. 9 CINE datasets were acquired with echo times ranging from 2.04ms to 10.20ms with a interleaved multi-shot multi-echo gradient echo technique over three breath holds. Other imaging parameters were set to: flip angle=20°, acquisition data matrix=256x224, FOV=(288x252)mm2, in-plane resolution=(1.1x1.1)mm2, slice thickness=4mm and acceleration using GRAPPA (R=3). Prior to T2* mapping, volume selective B0 shimming was conducted to reduce static magnetic field inhomogeneities.

## Results

**Figure 1:**
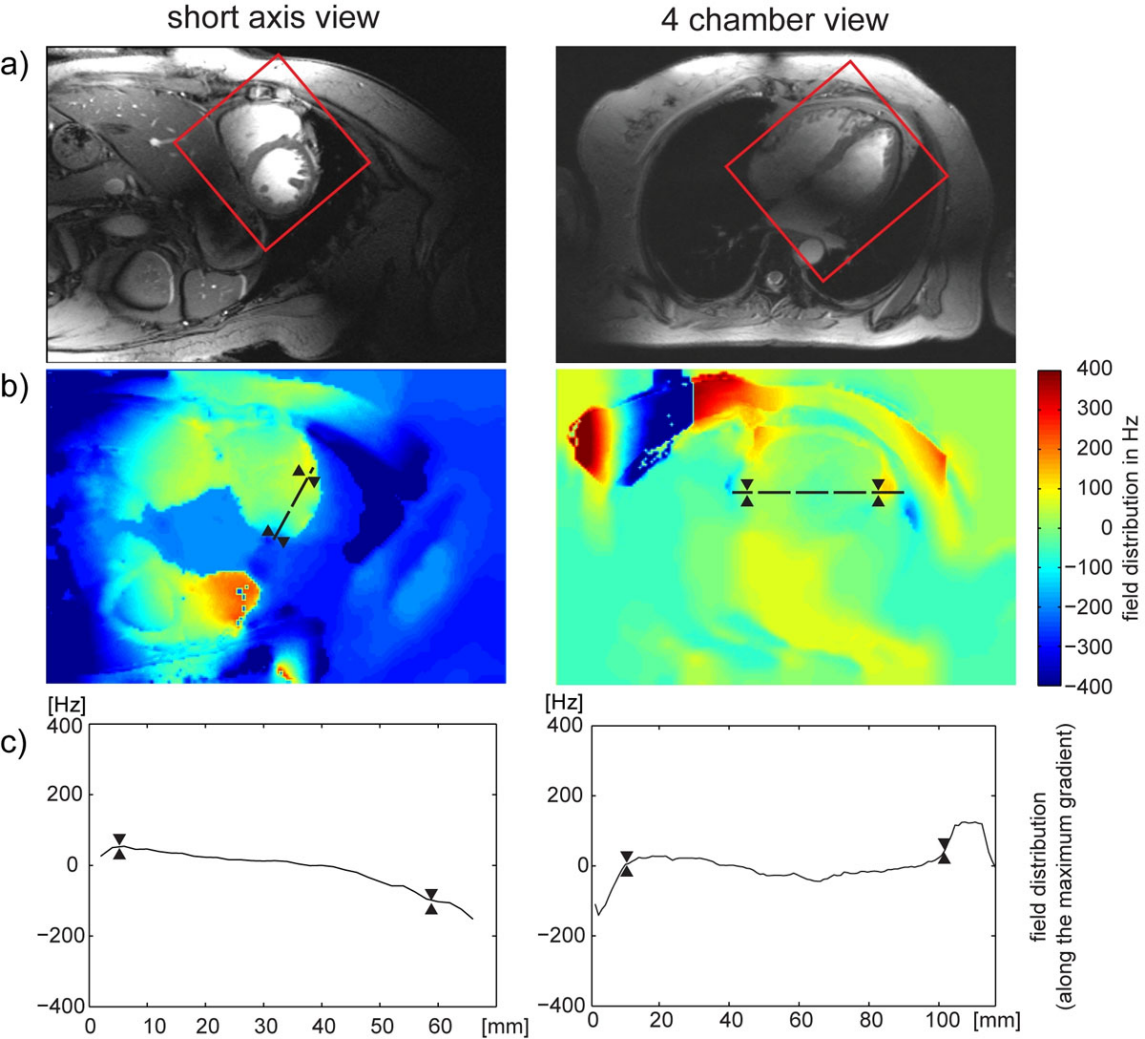
A) Short axis view and four chamber view of the heart illustrating the positioning of the volume (marked in red) used for volume selective shimming. B) B0 field variation derived after volume selective shimming. For this subject the global shim provided a peak-to-peak field variation was of about 400 Hz across the entire heart. After volume selective shimming peak-to-peak B0 variation across the heart was reduced to approximately 80 Hz. The direction of the maximal B0 gradient is illustrated by the dashed black line in and the corresponding profile of B0 field distribution is plotted in C). To guide the eye the epicardial borders are marked in B) and C) by two triangles.

After volume selective shimming, a mean peak-to-peak B0 difference of approximately 80 Hz was found across the entire heart for a four chamber view and a mid-ventricular short axis view of the heart (Fig1). The through plane field gradient at the myocardium/epicardial fat/lung interface was found to be much more pronounced versus the through-plane field gradient obtained for the left and right ventricle as illustrated in Fig. 1. The latter showed a mean of 3 Hz/mm, which translates into an through-plane B0 dispersion of approximately 12 Hz/voxel for a 4 mm slice thickness. This B0 gradient implies that macroscopic intravoxel dephasing effects are of minor effect for the TE range used.

**Figure 2:**
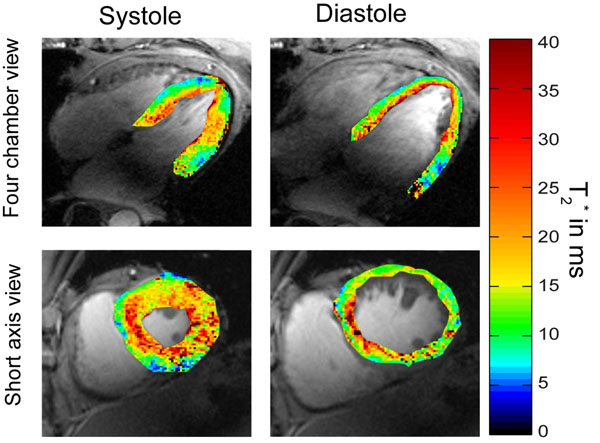
Four chamber (top) and short axis view T2* colour maps obtained from CINE T2* Mapping superimposed to anatomical 2D CINE FLASH gray scale images. Systolic and diastolic T2* maps show significant differences.

No severe susceptibility artifacts were detected in the septum and in the lateral wall for T2* weighting. For TE>7ms, a signal void (related to susceptibility weighting) was observed within the anterior and inferior myocardial segments. The longest T2* values were found for anterior (T2*=14.0 ms), anteroseptal (T2*=17.2 ms) and inferoseptal (T2*=16.5 ms) myocardial segments. Shorter T2* values were observed for inferior (T2*=10.6 ms) and inferolateral (T2*=11.4 ms) segments. A significant difference (p=0.002) in T2* values was observed between end-diastole and end-systole as illustrated in Figure 2. T2* changes of up to approximately 27% were observed across the cardiac cycle. Cardiac cycle dependent T2* changes were pronounced in the septum.

## Conclusions

Our results underscore the challenges of myocardial T2* mapping at 7.0 T due to the propensity to macroscopic susceptibility artefacts and T2* shortening, but demonstrate that these issues can be offset by using tailored shimming techniques together with dedicated acquisition schemes.

